# Hyperbolic systems with non-diagonalisable principal part and variable multiplicities, I: well-posedness

**DOI:** 10.1007/s00208-018-1672-1

**Published:** 2018-03-22

**Authors:** Claudia Garetto, Christian Jäh, Michael Ruzhansky

**Affiliations:** 1 Department of Mathematical Sciences, Loughborough University, Loughborough, Leicestershire LE11 3TU UK; 2 Department of Mathematics, Imperial College London, 180 Queen’s Gate, London, SW7 2AZ UK

**Keywords:** 35L45 (primary), 46E35 (secondary)

## Abstract

In this paper we analyse the well-posedness of the Cauchy problem for a rather general class of hyperbolic systems with space-time dependent coefficients and with multiple characteristics of variable multiplicity. First, we establish a well-posedness result in anisotropic Sobolev spaces for systems with upper triangular principal part under interesting natural conditions on the orders of lower order terms below the diagonal. Namely, the terms below the diagonal at a distance *k* to it must be of order $-\,k$. This setting also allows for the Jordan block structure in the system. Second, we give conditions for the Schur type triangularisation of general systems with variable coefficients for reducing them to the form with an upper triangular principal part for which the first result can be applied. We give explicit details for the appearing conditions and constructions for $2\times 2$ and $3\times 3$ systems, complemented by several examples.

## Introduction

The main aim of this paper is to consider the Cauchy problem for hyperbolic systems

1 $$\begin{aligned} \left\{ \begin{array}{l@{\quad }l} D_t u = A(t,x,D_x)u + B(t,x,D_x)u + f(t,x), &{} \quad (t,x) \in [0,T] \times \mathbb R^n, \\ \left. u\right| _{t=0} = u_0, &{} \quad x \in \mathbb R^n, \end{array} \right. \end{aligned}$$

with the usual notation $D_t=-\,\mathrm{i}\partial _t$ and $D_x=-\,\mathrm{i}\partial _x$. Here, we assume that $A(t,x,D_x) = \big [ a_{ij}(t,x,D_x) \big ]_{i,j=1}^m$ is an $m\times m$ matrix of pseudo-differential operators of order 1, i.e. $a_{ij} \in C([0,T],\Psi _{1,0}^1(\mathbb R^n))$ with possibly complex valued symbols. In the first part of the paper we will also assume that

2 $$\begin{aligned} A(t,x,D_x) = \Lambda (t,x,D_x) + N(t,x,D_x), \end{aligned}$$

with real-valued symbols in

$$\begin{aligned} \Lambda (t,x,D_x) = {{\mathrm{diag}}}(\lambda _1(t,x,D_x),\lambda _2(t,x,D_x),\ldots ,\lambda _m(t,x,D_x)), \end{aligned}$$

and

$$\begin{aligned} N(t,x,D_x) = \begin{bmatrix} 0&\quad a_{12}(t,x,D_x)&\quad a_{13}(t,x,D_x)&\quad \cdots&\quad a_{1m}(t,x,D_x) \\ 0&\quad 0&\quad a_{23}(t,x,D_x)&\quad \cdots&\quad a_{2m}(t,x,D_x) \\ \vdots&\quad \vdots&\quad \vdots&\quad \cdots&\quad \vdots \\ 0&\quad 0&\quad 0&\quad \ldots&\quad a_{m-1m}(t,x,D_x)\\ 0&\quad 0&\quad 0&\quad \ldots&\quad 0 \end{bmatrix}. \end{aligned}$$

Finally, we assume that

$$\begin{aligned} B(t,x,D_x) = \big [ b_{ij}(t,x,D_x) \big ]_{i,j=1}^m, \quad b_{ij} \in C([0,T],\Psi _{1,0}^0(\mathbb R^n)), \end{aligned}$$

is an $m\times m$ matrix of pseudo-differential operators of order 0 with possibly complex valued symbols. We can take any $n\ge 1$ and we can assume that $m\ge 2$ since in the case $m=1$ there are no multiplicities and thus much more is known. It is also well-known that even if all the coefficients in *A* and *B* depend only on time, due to multiplicities, the best one can hope for is the well-posedness of the Cauchy problem (1) in suitable classes of Gevrey spaces. Thus, the main questions that we address in this paper are:

1. Under what structural conditions on the zero order part $B(t,x,D_x)$ is the Cauchy problem (1) well-posed in $C^\infty$ or, even better, in suitable scales of Sobolev spaces?
2. Under what conditions on the general matrix $A(t,x,D_x)$ of first order pseudo-differential operators can we reduce it (microlocally) to another system with *A* satisfying the upper triangular condition (2)?

Note that this paper is part of a wider analysis of hyperbolic systems with multiplicities. Here we investigate the well-posedness of these systems. In the second part of this paper we plan to carry out the microlocal analysis of their solutions.

In the case of $2\times 2$ systems the questions above have been analysed with the answer to (Q1) given by the following theorem:

### Theorem A

([27, Theorem 7.2]) Let $m=2$. Suppose that the pseudo-differential operator ${b}_{21}$ is of order not greater than $-\,1$. Then the Cauchy problem (1) is well-posed in $C^\infty$. Moreover, it is well-posed in the anisotropic Sobolev space $$\begin{bmatrix} H^{s_1}(\mathbb R^n) \\ H^{s_2}(\mathbb R^n) \end{bmatrix}$$ provided $s_2-s_1 \ge 1$. In that case the solution satisfies the following estimates:

$$\begin{aligned} \Vert u_1(t,\cdot )\Vert _{H^s} + \Vert u_2(t,\cdot )\Vert _{H^{s+1}} \le c e^{ct} \left( \Vert u_1^0\Vert _{H^s} + \Vert u_2^0\Vert _{H^{s+1}} \right) , \quad 0 \le t \le T, \end{aligned}$$

for $u_j^0 \in H_{comp}^{s+j-1}(\mathbb R^n)$, $j=1,2$, with $c>0$ depending on *s*, *T*, and the support of the initial data.

The case of systems of general size but for coefficients depending only on *t* and for $n=1$ was also considered. More precisely, in [26] the authors considered the Cauchy problem

3 $$\begin{aligned} \left\{ \begin{array}{l@{\quad }l} D_t u = A(t)D_x u + B(t)D_x u + f(t,x), &{} \quad (t,x) \in [0,T] \times \mathbb R, \\ \left. u\right| _{t=0} = 0, &{} \quad x \in \mathbb R, \end{array} \right. \end{aligned}$$

with $A(t) = \big [ a_{ij}(t) \big ]_{i,j=1}^m \in C([0,T])^{m \times m}$ in the form

$$\begin{aligned} A(t) = \Lambda (t) + N(t), \end{aligned}$$

similar to (2). They showed the following result in the absence of lower order terms and for zero Cauchy data:

### Theorem B

([26, Proposition 1]) Let $B(t) \equiv 0$ and let $s \in \mathbb R$. Then the Cauchy problem (3) is $C^\infty$-well-posed. Moreover, there exist $r_1$,..., $r_{m-1} \in [0,1]$ such that for every $f \in C(\mathbb R, (H^s(\mathbb R))^m)$ identically 0 at $t=0$ it admits a unique solution $u \in C(\mathbb R,({\mathcal {S}}'(\mathbb R))^m)$ satisfying

$$\begin{aligned} u_m \in C(\mathbb R, H^s(\mathbb R)), \quad u_{m-j} \in C(\mathbb R, H^{s-r_1-\cdots -r_{j-1}}(\mathbb R)), \end{aligned}$$

for $j = 1, \ldots , m-1$, and identically 0 at $t=0$. In particular, if $\lambda _j(t) \ne \lambda _k(t)$, $t \in \mathbb R$, $1 \le j < k \le m$, no loss of anisotropic regularity appears.

The case of (microlocally) *diagonalisable* systems of any order with fully variable coefficients was considered by Rozenblum [41] under the condition of transversality of the intersecting characteristics. Also allowing the variable multiplicities, this transversality condition was later removed in [32, 33] with sharp $L^p$-estimates for solutions, with further applications to the spectral asymptotics of the corresponding elliptic systems.

Before stating our main results and collecting some necessary basic notions we give a brief overview of the state of the art for hyperbolic equations and systems. We have a complete understanding of strictly hyperbolic systems, i.e., systems without multiplicities, with $C^\infty$-coefficients. This starts with the groundbreaking work of Lax [35] and Hörmander [28] and heavily relies on the modern theory of Fourier integral operators (FIO). Well-posedness is here obtained in the space of distributions ${\mathcal {D}}'$. There are also well-posedness results for less regular coefficients with respect to *t*. For instance, well-posedness with loss of derivatives has been obtained by Colombini and Lerner [9] for second order strictly hyperbolic equations with Log-Lipschitz coefficients with respect to *t* and smooth in *x*. It is possible to further drop the regularity in *t* (for instance Hölder), however, this has to be balanced by stronger regularity in *x* (Gevrey) and leads to more specific (Gevrey) well-posedness results (see [3, 31] and references therein). Paradifferential techniques have been recently used for this kind of strictly hyperbolic equations by Colombini et al. [6, 7].

The analysis of hyperbolic equations with multiplicities (weakly hyperbolic) has started with the seminal paper by Colombini et al. [5] in the case of coefficients depending only on time. Profound difficulties in such analysis have been exhibited by Colombini et al. [4, 8] showing that even the second order wave equation in ${\mathbb {R}}$ with smooth time-dependent propagation speed (but with multiplicity) and smooth Cauchy data need not be well-posed in ${\mathcal {D}}'$. However, they turn out to be well-posed in suitable Gevrey classes or spaces of ultradistributions. In the last decades many results were obtained for weakly hyperbolic equations with *t*-dependent coefficients ([3, 11, 16, 18–20, 34], to quote only very few). More recently, advances in the theory of weakly hyperbolic systems with *t*-dependent coefficients have been obtained for systems of any size in presence of multiplicities with regular or low regular (Hölder) coefficients [16, 22, 23]. In addition, in [17] precise conditions on the lower order terms (Levi conditions) have been formulated to guarantee Gevrey and ultradistributional well-posedness. Previously very few results were known in the field for systems of a certain size ($2\times 2$, $3\times 3$) [12, 13] or of a certain form (for instance without lower order terms or with principal part of a certain form) [44].

Weakly hyperbolic equations with *x*-dependent coefficients were considered for the first time in the celebrated paper by Bronshtein [2]. As shown already in some earlier works by Ivrii, the corresponding Cauchy problem is well-posed under “almost analytic regularity”, namely, if the coefficients and initial data are in suitable Gevrey classes. Bronshtein’s result was extended to (*t*, *x*)-dependent scalar equations by Ohya and Tarama [38] and to systems by Kajitani and Yuzawa [31]. The regularity assumptions are always quite strong with respect to *x* (Gevrey) and not below Hölder in *t*. See also [10, 37]. Geometrical and microlocal analytic approaches are known for equations or systems under specific assumptions on the characteristics and/or lower order terms. See [29, 30, 33, 36, 39], to quote only a few. Time-dependent coefficients of low regularity (distributional) have been considered in [21].

In this paper we will be interested in the case of coefficients depending on both *t* and *x* and we will make use of the usual definitions of symbol classes. We say that a (possibly) complex valued function $a=a(x,\xi ) \in C^{\infty }(\mathbb R^n \times \mathbb R^n)$ belongs to $S^m_{1,0}( \mathbb R^n \times \mathbb R^n)$ if there exist constants $C_{\alpha ,\beta } >0$ such that

$$\begin{aligned} \forall \alpha ,\beta \in \mathbb N_0^n \,\, : \,\, |\partial _x^\alpha \partial _\xi ^\beta a(x,\xi )| \le C_{\alpha ,\beta } \left\langle \xi \right\rangle ^{m-|\beta |} \quad \forall (x,\xi ) \in \mathbb R^n \times \mathbb R^n. \end{aligned}$$

The set of pseudo-differential operators associated to the symbols in $S^m_{1,0}( \mathbb R^n \times \mathbb R^n)$ is denoted by $\Psi ^m_{1,0}( \mathbb R^n \times \mathbb R^n)$.

If there is no question about the domain under consideration, we will abbreviate the symbol- and operator-classes by $S^m_{1,0}$ and $\Psi ^m_{1,0}$, respectively, or simply by $S^m$ and $\Psi ^m$.

We also denote by $C([0,T], S_{1,0}^m(\mathbb R^n \times \mathbb R^n))$ the space of all symbols $a(t,x,\xi )\in S_{1,0}^m(\mathbb R^n\times \mathbb R^n)$ which are continuous with respect to *t*. The set of operators associated to the symbols in $C([0,T], S_{1,0}^m(\mathbb R^n \times \mathbb R^n))$ is denoted by $C([0,T], \Psi _{1,0}^m(\mathbb R^n \times \mathbb R^n))$.

Again, if there is no question about the domain under consideration, we will abbreviate the symbol- and operator-classes by $C S_{1,0}^m$ and $C\Psi ^m_{1,0}$, respectively, or simply by $C S^m$ and $C\Psi ^m$.

Let us give our main result concerning the first question (Q1) for the systems with the principal part *A* satisfying the upper triangular condition (2). Here, $f_k$, $u_k$ and $u_k^0$, for $k=1,\ldots ,m$, stand for the components of the vectors *f*, *u* and $u_0$, respectively.

### Theorem 1

Let $n\ge 1$, $m\ge 2$, and let

4 $$\begin{aligned} \left\{ \begin{array}{l@{\quad }l} D_t u = A(t,x,D_x)u + B(t,x,D_x)u + f(t,x), &{}\quad (t,x) \in [0,T] \times \mathbb R^n, \\ \left. u \right| _{t=0} = u_0(x), &{}\quad x \in \mathbb R^n, \end{array} \right. \end{aligned}$$

where $A(t,x,D_x) \in (CS^1)^{m \times m}$ is an upper-triangular matrix of pseudo-differential operators of order 1 in the form (2), and $B(t,x,D_x) \in (CS^0)^{m \times m}$ is a matrix of pseudo-differential operators of order 0, continuous with respect to *t*. Hence, if

$$\begin{aligned} \hbox {the lower order terms } b_{ij} \hbox { belong to } C([0,T], \Psi ^{j-i}) \hbox { for } i> j, \end{aligned}$$

$u^0_k\in H^{s+k-1}(\mathbb R^n)$ and $f_k\in C([0,T],H^{s+k-1})$ for $k=1,\ldots ,m$, then (4) has a unique anisotropic Sobolev solution *u*, i.e., $u_k\in C([0,T], H^{s+k-1})$ for $k=1,\ldots , m$.

### Remark 1

As stated earlier, we allow *A* and *B* to have complex valued symbols as long as the symbols of $\Lambda$ in (2), i.e. the eigenvalues of $A(t,x,\xi )$, are real valued.

The main condition of Theorem 1 for the Sobolev well-posedness is that the pseudo-differential operator $b_{ij}$ below the diagonal (i.e. for $i>j$) must be of order $j-i$. In other words, the terms below the diagonal at a distance *k* to it must be of order $-\,k$.

In solving the Cauchy problem (4) we will make use of Fourier integral operators depending on the parameter $t\in [0,T]$. Namely, we will work with operators of the type

$$\begin{aligned} \int \limits _{0}^{t} \int \limits _{\mathbb R^n} e^{\mathrm{i}\varphi (t,s,x,\xi )} a(t,s,x,\xi ) {\widehat{g}}(s,\xi ) d\xi ds \end{aligned}$$

where $\varphi$ is the solution of a certain eikonal equation and the symbol *a* is determined via asymptotic expansion and transport equations. In Sect. 2.1 we will recall some well-known Sobolev estimates for this type of operators.

In Sect. 2 we will prove Theorem 1 after we explain its idea in the cases of $m=2$ and $m=3$.

Consequently, in Sect. 3 we give an answer to the second question (Q2) above in the form of a suitable variable coefficients extension of the Schur triangularisation. For constant matrices such a procedure is well known (see e.g. [1, Theorem 5.4.1]).

### Theorem C

(Schur’s triagularisation theorem) Given a (constant) $m \times m$ matrix *A* with eigenvalues $\lambda _1, \ldots , \lambda _m$ in any prescribed order, there is a unitary $m \times m$ matrix *T* such that $R = T^{-1} A T$ is upper triangular with the diagonal elements $r_{ii} = \lambda _i$. Furthermore, if the entries of *A* and its eigenvalues are all real, *T* may be chosen to be real orthogonal.

It follows that *R* can be written as $D+N$, where $D={{\mathrm{diag}}}(\lambda _1,\ldots ,\lambda _m)$ and *N* is a nilpotent upper triangular matrix.

If the matrix *A* depends on one or several parameters, namely $A=A(t,x,\xi )$, the situation becomes less clear and it is difficult to give a complete description, in particular together with a prescribed regularity of the involved transformation matrices. The regularity of the matrix *A* and the desire to maintain it through the transformation puts already constrains on the matrix as, in general, the eigenvalues can only be expected to be Lipschitz continuous in the parameters even if all the entries depend smoothly on the parameters (see, e.g., [2, 40] and the references therein). In the sequel, we will present some sufficient conditions to ensure the existence of an upper triangularisation for $A(t,x,\xi )$ which respects its regularity. For example, it will apply to the case when *A* is a matrix of first order symbols continuous with respect to *t*, i.e., $A(t,x,\xi ) \in \big ( C S^1 \big )^{m \times m}$.

Our main result for this part of the problem is the following theorem.

### Theorem 2

Let $A(t,x,\xi ) \in (C S^1)^{m \times m}$, be a $m \times m$-matrix with eigenvalues $\lambda _1, \ldots , \lambda _{m} \in C S^1$, and let $h_1 , \ldots , h_{m-1} \in \big ( C S^0 \big )^m$ be the corresponding eigenvectors. Suppose that for $e_1=\big [1,0,\ldots ,0 \big ]^T \in \mathbb R^{m-i+1}$ the condition

5 $$\begin{aligned} \left\langle h^{(i)}(t,x,\xi ) | e_1 \right\rangle \ne 0, \quad \forall (t,x,\xi ) \in [0,T] \times \mathbb R^n \times \mathbb R^n \end{aligned}$$

holds for all $i=1,\ldots , m-1$, with the notation for $h^{(i)}$ explained in (37). Then, there exists a matrix-valued symbol $T(t,x,\xi ) \in (C S^0)^{m \times m}$, invertible for $(t,x,\xi ) \in [0,T] \times \mathbb R^n \times \{ |\xi | \ge M\}$ with $T^{-1}(t,x,\xi ) \in (CS^0)^{m \times m}$, such that

$$\begin{aligned} T^{-1}(t,x,\xi )A(t,x,\xi ) T(t,x,\xi ) = \Lambda (t,x,\xi ) + N(t,x,\xi ) \end{aligned}$$

for all $(t,x,\xi ) \in [0,T] \times \mathbb R^n \times \{ |\xi | \ge M \}$, where

$$\begin{aligned} \Lambda (t,x,\xi ) = {{\mathrm{diag}}}(\lambda _1(t,x,\xi ),\lambda _2(t,x,\xi ),\ldots ,\lambda _m(t,x,\xi )) \end{aligned}$$

and

$$\begin{aligned} N(t,x,\xi ) = \begin{bmatrix} 0&\quad N_{12}(t,x,\xi )&\quad N_{13}(t,x,\xi )&\quad \cdots&\quad N_{1m}(t,x,\xi ) \\ 0&\quad 0&\quad N_{23}(t,x,\xi )&\quad \cdots&\quad N_{2m}(t,x,\xi ) \\ \vdots&\quad \vdots&\quad \vdots&\quad \cdots&\quad \vdots \\ 0&\quad 0&\quad 0&\quad \ldots&\quad N_{m-1m}(t,x,\xi )\\ 0&\quad 0&\quad 0&\quad \ldots&\quad 0 \end{bmatrix}, \end{aligned}$$

and *N* is a nilpotent matrix with entries in $C S^1$.

Furthermore, there is an expression for the matrix symbol *T* which will be given in Theorem 6. Also, the assumption (5) can be relaxed, see Remark 6. In Sect. 3 we will prove this result as well as describe the procedure how to obtain the desired upper triangular form. Moreover, we work out in detail the cases of $m=2$ and $m=3$ clarifying this Schur triangualisation procedure and give a number of examples.

The results and techniques of this paper are a natural outgrowth of the paper [27] where the case $m=2$ was considered and to which the results of the present paper reduce in the case of $2\times 2$ systems. It is with great sorrow that we remember the untimely departure of our colleague and friend **Todor Gramchev** who was the inspiration for both [27] and the present paper.

## Well-posedness in anisotropic Sobolev spaces

This section is devoted to proving the well-posedness of the Cauchy problem (1). For the reader’s convenience we first give a detailed proof in the cases $m=2$ and $m=3$. This will inspire us in proving Theorem 1. We note that the case $m=2$ has been studied in [27] and we will briefly review its derivartion. However, first we collect a few results about Fourier integral operators that we will need in the sequel.

### Auxiliary remarks

In solving the Cauchy problem (1), we will deal with solutions of certain scalar pseudo-differential equations. For each characteristic $\lambda _j$ of *A*, we will be denoting by $G^0_j\theta$ the solution to

$$\begin{aligned} \left\{ \begin{array}{l} D_t w = \lambda _j(t,x,D_x)w + b_{jj}(t,x,D_x)w, \\ w(0,x) = \theta (x), \end{array}\right. \end{aligned}$$

and by $G_j g$ the solution to

$$\begin{aligned} \left\{ \begin{array}{l} D_t w = \lambda _j(t,x,D_x)w + b_{jj}(t,x,D_x)w + g(t,x), \\ w(0,x) = 0. \end{array}\right. \end{aligned}$$

The operators $G^0_j$ and $G_j$ can be microlocally represented by Fourier integral operators

6 $$\begin{aligned} G^0_j \theta (t,x) = \int \limits _{\mathbb R^n} e^{\mathrm{i}\varphi _j(t,x,\xi )} a_j(t,x,\xi ) {\widehat{\theta }}(\xi ) d\xi \end{aligned}$$

and

$$\begin{aligned} G_j g(t,x) = \int \limits _{0}^{t} \int \limits _{\mathbb R^n} e^{\mathrm{i}\varphi _j(t,s,x,\xi )} A_j(t,s,x,\xi ) {\widehat{g}}(s,\xi ) d\xi ds, \end{aligned}$$

with $\varphi _j(t,s,x,\xi )$ solving the eikonal equation

$$\begin{aligned} \left\{ \begin{array}{l} \partial _t \varphi _j = \lambda _j(t,x,\nabla _x\varphi _j), \\ \varphi _j(s,s,x,\xi ) = x \cdot \xi , \end{array} \right. \end{aligned}$$

and with the notation

$$\begin{aligned} \varphi _j(t,x,\xi ) = \varphi _j(t,0,x,\xi ). \end{aligned}$$

Here we also have the amplitudes $A_{j,-k}(t,s,x,\xi )$ of order $-\,k$, *k* $\in \mathbb N$, giving $A_j \sim \sum _{k=0}^{\infty } A_{j,-k}$, and they satisfy the transport equations with initial data at $t=s$, and we have $a_j(t,x,\xi ) = A_j(t,0,x,\xi )$.

If $a_j\in S^m$, i.e. if the amplitude $a_j$ in (6) is a symbol of order *m*, we will write $G^0_j\in I_{1,0}^{m}.$ However, in the above construction of propagators for hyperbolic equations, we have $a_j\in S^0$, so that $G^0_j \in I_{1,0}^{0}$.

By $I^m_{1,0}$, we denote the class of Fourier integral operators with amplitudes in $S^m_{1,0}$. For further information, the reader may consult [15, 42, 43] and the references therein.

With that, we can record the following estimate:

#### Lemma 1

For any $\sigma \in {\mathbb {R}}$, for sufficiently small *t*, we have

$$\begin{aligned} \left\| G_j^0\theta (t) \right\| _{H^\sigma } \le C_{A,\sigma ,u_0} \Vert \theta \Vert _{H^\sigma },\quad \left\| G_j g(t) \right\| _{H^\sigma } \le C_{A,\sigma } t \Vert g\Vert _{L^\infty _s H^\sigma _x}. \end{aligned}$$

This statement follows from the continuity of $\lambda _j, \varphi _j, a_j, A_j$ with respect to *t* and from the $H^\sigma$-boundedness of non-degenerate Fourier integral operators, see e.g. [15] (there are also surveys on such questions [42, 43]). It is important to note that the constant for the estimate for $G_j$ does not depend on the initial data of the Cauchy problem; see also Remark 2.

### The case $m=2$

To motivate the higher order cases, here we review the construction for $2\times 2$ systems adapting it for the subsequent higher order arguments. Hence, in this subsection we follow the proof in [27]. Thus, we consider the system

7 $$\begin{aligned} \left\{ \begin{array}{l@{\quad }l} D_t u = A(t,x,D_x)u + B(t,x,D_x)u + f(t,x), &{}\quad (t,x) \in [0,T] \times \mathbb R^n,\\ \left. u\right| _{t=0} = u_0, &{}\quad x \in \mathbb R^n, \end{array} \right. \end{aligned}$$

where $u_0(x) = \big [u_1^0(x),u_2^0(x) \big ]^T$, $f(t,x) = \big [ f_1(t,x) ,f_2(t,x) \big ]^T$, and with the operators $A(t,x,D_x)$ and $B(t,x,D_x)$ given by

8 $$\begin{aligned} A(t,x,D_x) = \begin{bmatrix} \lambda _1(t,x,D_x)&\quad a_{12}(t,x,D_x) \\ 0&\quad \lambda _2(t,x,D_x) \\ \end{bmatrix} \end{aligned}$$

and

$$\begin{aligned} B(t,x,D_x) = \begin{bmatrix} b_{11}(t,x,D_x)&\quad b_{12}(t,x,D_x) \\ b_{21}(t,x,D_x)&\quad b_{22}(t,x,D_x) \\ \end{bmatrix}. \end{aligned}$$

We suppose that all entries of $A(t,x,D_x)$ belong to $C \Psi _{1,0}^1$ and all entries of $B(t,x,D_x)$ belong to $C \Psi _{1,0}^0$. By using the operators $G^0_j$ and $G_j$ introduced in Sect. 2.1, we can reformulate the Eq. (7) as

9 $$\begin{aligned} u_1= & {} U^0_1 + G_1((a_{12}+b_{12})u_2), \end{aligned}$$

10 $$\begin{aligned} u_2= & {} U^0_2 + G_2(b_{21}u_1), \end{aligned}$$

where

11 $$\begin{aligned} U_j^0 = G_j^0 u_j^0 + G_j(f_j), \quad j=1,2. \end{aligned}$$

Plugging (10) in (9), we obtain

12 $$\begin{aligned} u_1 = {\tilde{U}}^0_1 + G_1(a_{12}G_2(b_{21}u_1)) + G_1(b_{12}G_2(b_{21}u_1)), \end{aligned}$$

where

13 $$\begin{aligned} {\tilde{U}}^0_1 = G_1^0 u_1^0 + G_1(f_1) + G_1((a_{12}+b_{12})U^0_2). \end{aligned}$$

Using the rules of composition of Fourier integral operators, see e.g. [15], and by Lemma 1, we get that the operator $G_1 \circ a_{12} \circ G_2 \circ b_{21}$ in (12) acts continuously on $H^s$ if it is of order 0. Since $a_{12} \in C \Psi _{1,0}^1$ we therefore need to assume that $b_{21} \in C \Psi _{1,0}^{-1}$.

The operator $$G_1 \circ b_{12} \circ G_2 \circ b_{21}$$ belongs to $$ C I_{1,0}^{-1}$$ since $$b_{21} \in C \Psi _{1,0}^{-1}$$ and $$b_{12} \in C \Psi _{1,0}^{0}$$.

We now introduce the following scale of Banach spaces $X^s(t) := C([0,t],H^s)$, $t \in [0,T]$, equipped with the norm

$$\begin{aligned} \Vert u\Vert _{X^s(t)} = \sup _{\tau \in [0,t]} \Vert u(\tau ,\cdot )\Vert _{H^s}. \end{aligned}$$

Let

$$\begin{aligned} \mathcal G_1^0 u_1:= G_1(a_{12}G_2(b_{21}u_1)) + G_1(b_{12}G_2(b_{21}u_1)). \end{aligned}$$

It follows that (12) can be written as

$$\begin{aligned} u_1={\tilde{U}}^0_1 +\mathcal G_1^0 u_1. \end{aligned}$$

By composition of Fourier integral operators and Lemma 1 we have that the 0-order Fourier integral operator $$\mathcal G_1^0$$ maps $$ C([0,T],H^s)$$ continuously into itself and for small time interval it is a contraction, in the sense that there exists $$T^*\in [0,T]$$ such that

$$\begin{aligned} \Vert {\mathcal {G}}_1^0(u-v)\Vert _{X^s(T^*)} \le C_{A,s} T^*\Vert u-v\Vert _{X^s(T^*)}, \end{aligned}$$

with $$C_{A,s}T^*< 1$$. Banach’s fixed point theorem ensures the existence of a unique fixed point $$u_1$$ for the map $$\mathcal G_1^0$$. Hence, by assuming that the initial data $${\tilde{U}}^0_1$$ belongs to $$ C([0,T^*],H^s)$$ we conclude that there exists a unique $$u_1\in C([0,T^*],H^s) $$ solving (12). Note that the same argument proves that the operator $$I-{\mathcal {G}}_1^0$$ is invertible on a sufficiently small interval in *t* since $${\mathcal {G}}_1^0=I$$ at $$t=0$$. From formula (13) it is clear that in order to get $${\tilde{U}}^0_1$$ to belong to $$ C([0,T^*],H^s)$$ we need to assume that $$U^0_2\in H^{s+1}$$. Finally, we get $$u_2$$ by substitution of $$u_1$$ in (10).

#### Remark 2

Note that the constant $T^*$ depends only on *A* and *s*. Thus, the argument above can be iterated by taking $u(T^*,x)$ as new initial data. In this way one can cover an arbitrary finite interval [0, *T*] and obtain a solution in $C([0,T],H^s)\times C([0,T],H^{s+1})$.

#### Remark 3

Since $a_{12}(t,x,D_x)$ is a first order operator combining (11) with (13) we easily see that in order to get Sobolev well-posedness of order *s* we need to take initial data $u_1^0$ and $u_2^0$ in $H^s$ and $H^{s+1}$, respectively, and right hand-side functions $f_1$ and $f_2$ in $C([0,T],H^s)$ and $C([0,T],H^{s+1})$, respectively.

We have therefore proved the following theorem stated for the first time in [27, Theorem 7.2].

#### Theorem 3

Consider the Cauchy problem (7), with the $2 \times 2$ matrices

$$\begin{aligned} A(t,x,D_x) \in (CS^1)^{2 \times 2} \quad \text {and} \quad B(t,x,D_x) \in (CS^0)^{2 \times 2}, \end{aligned}$$

where *A* is of the form (2). Assume that ${b}_{21} \in C([0,T],\Psi ^{-1}_{1,0})$, the right hand-side functions $f_1$ and $f_2$ belong to $C([0,T],H^s)$ and $C([0,T],H^{s+1})$, respectively, and the initial data $u_1^0$ and $u_2^0$ belong to $H^s$ and $H^{s+1}$, respectively. Then, (7) has a unique solution in $C([0,T],H^s)\times C([0,T],H^{s+1})$. More generally it is well-posed in the anisotropic Sobolev space $C([0,T],H^{s_1})\times C([0,T],H^{s_2})$, provided $s_2-s_1 = 1$.

#### Remark 4

It was also shown in [27] that the solution *u* satisfies the estimate

$$\begin{aligned} \Vert u_1(t,\cdot )\Vert _{H^s} + \Vert u_2(t,\cdot )\Vert _{H^{s+1}} \le c e^{ct} \left( \Vert u_1^0\Vert _{H^s} + \Vert u_2^0\Vert _{H^{s+1}} \right) , \quad 0 \le t \le T, \end{aligned}$$

for $u_j^0 \in H^{s+j-1}_{comp}$, $j=1,2$ with $c>0$ depending on *s*, *T*, and the support of the initial data. Since well-posedness is obtained for any Sobolev order *s* it follows that the Cauchy problem (7) is also $C^\infty$ well-posed.

### The case $m=3$

In this section we will extend the construction to the case of $3\times 3$ systems. In the argument there is an additional substitution and a fixed point argument step compared to the case $m=2$. The advantage of giving the case of $m=3$ here is that we can make the argument more concrete compared to the more abstract construction in the general case that will be given in the following section. Thus, let

14 $$\begin{aligned} \left\{ \begin{array}{l@{\quad }l} D_t u = A(t,x,D_x)u + B(t,x,D_x)u + f(t,x), &{}\quad (t,x) \in [0,T] \times \mathbb R^n,\\ \left. u\right| _{t=0} = u_0, &{}\quad x \in \mathbb R^n, \end{array} \right. \end{aligned}$$

where $u_0(x) = \big [u_1^0(x),u_2^0(x),u_3^0(x) \big ]^T$, $f(t,x) = \big [ f_1(t,x) ,f_2(t,x),f_3(t,x) \big ]^T$, $A(t,x,D_x)$ is defined by the matrix

15 $$\begin{aligned} \begin{bmatrix} \lambda _1(t,x,D_x)&\quad a_{12}(t,x,D_x)&\quad a_{13}(t,x,D_x) \\ 0&\quad \lambda _2(t,x,D_x)&\quad a_{23}(t,x,D_x) \\ 0&\quad 0&\quad \lambda _3(t,x,D_x) \end{bmatrix}, \end{aligned}$$

and

$$\begin{aligned} B(t,x,D_x) = \begin{bmatrix} b_{11}(t,x,D_x)&\quad b_{12}(t,x,D_x)&\quad b_{13}(t,x,D_x) \\ b_{21}(t,x,D_x)&\quad b_{22}(t,x,D_x)&\quad b_{23}(t,x,D_x) \\ b_{31}(t,x,D_x)&\quad b_{32}(t,x,D_x)&\quad b_{33}(t,x,D_x) \end{bmatrix}. \end{aligned}$$

We assume that all the entries of $A(t,x,D_x)$ and $B(t,x,\xi )$ belong to $C \Psi _{1,0}^1$ and $C \Psi _{1,0}^0$, respectively. Using the notations introduced earlier, we can write

16 $$\begin{aligned} \begin{aligned} u_3(t,x)&= U^0_3 + G_3(b_{31}u_1) + G_3(b_{32}u_2), \\ u_2(t,x)&= U^0_2 + G_2((a_{23}+b_{23})u_3) + G_2(b_{21}u_1), \\ u_1(t,x)&= U^0_1 + G_1((a_{12}+b_{12})u_2) + G_1((a_{13}+b_{13})u_3), \end{aligned} \end{aligned}$$

where

17 $$\begin{aligned} U_j^0(t,x) = G_j^0( u_j^0) + G_j(f_j), \quad j=1,2,3. \end{aligned}$$

Now, we plug $u_3$ into $u_1$ and $u_2$ in formula (16) and, thus, obtain

18 $$\begin{aligned} \begin{aligned} u_2(t,x)&= {\widetilde{U}}_2^0 + G_2(b_{21}u_1) + G_2((a_{23}+b_{23})G_3(b_{31}u_1)) \\&\quad +\, G_2((a_{23}+b_{23})G_3(b_{32}u_2)), \\ u_1(t,x)&= {\widetilde{U}}_1^0 + G_1((a_{13}+b_{13})G_3(b_{31}u_1)) \\&\quad +\, G_1((a_{13}+b_{13})G_3(b_{32}u_2)) + G_1((a_{12}+b_{12})u_2), \end{aligned} \end{aligned}$$

where

$$\begin{aligned} {\widetilde{U}}_j^0 = U^0_j + G_j((a_{j3}+b_{j3})(t,x,D_x)U^0_3), \quad j=1,2. \end{aligned}$$

We introduce the operator ${\mathcal {G}}^0_2$ by setting

19 $$\begin{aligned} {\mathcal {G}}^0_2u_2:=G_2((a_{23}+b_{23})G_3(b_{32}u_2)) \end{aligned}$$

and in analogy with the case $m=2$ we define

$$\begin{aligned} L_2 u_2 := u_2 - {\mathcal {G}}^0_2 u_2. \end{aligned}$$

By Lemma 1 we have that for any *s*, $${\mathcal {G}}^0_2$$ has the operator norm in $$H^s$$ strictly less than 1 on a sufficiently small interval $$[0,T^*]$$, so $$L_2$$ is a perturbation of the identity operator. By the Neumann series it follows that $$L_2$$ is invertible as a continuous operator from $$ C([0,T^*],H^s)$$ to $$ C([0,T^*],H^s)$$. Noting now that

$$\begin{aligned} u_2-{\mathcal {G}}^0_2u_2=L_2u_2={\widetilde{U}}_2^0 + G_2(b_{21}u_1) + G_2((a_{23}+b_{23})G_3(b_{31}u_1)), \end{aligned}$$

we have that

$$\begin{aligned} u_2(t,x) = L^{-1}_2{\widetilde{U}}_2^0 + L^{-1}_2G_2((a_{23}+b_{23})G_3(b_{31}u_1)) + L^{-1}_2G_2(b_{21}u_1). \end{aligned}$$

Since this expression depends only on $u_1$, we can plug it into the formula for $u_1$ in (18) and obtain

$$\begin{aligned} \begin{aligned} u_{1}(t,x)&= {\widetilde{U}}_1^0 + G_1((a_{13}+b_{13})G_3(b_{31}u_1)) + \\&\quad + G_1((a_{13}+b_{13})G_3(b_{32}u_2)) + G_1((a_{12}+b_{12})u_2)\\&= {\widetilde{U}}_1^0 + G_1((a_{13}+b_{13})G_3(b_{31}u_1)) + \\&\quad + G_1((a_{13}+b_{13})G_3(b_{32}(L^{-1}_2{\widetilde{U}}_2^0)) \\&\quad + G_1((a_{13}+b_{13})G_3(b_{32} L^{-1}_2G_2((a_{23}+b_{23})G_3(b_{31}u_1))))\\&\quad + G_1((a_{13}+b_{13})G_3(b_{32}(L^{-1}_2G_2(b_{21}u_1)))\\&\quad + G_1((a_{12}+b_{12})L^{-1}_2{\widetilde{U}}_2^0)\\&\quad + G_1((a_{12}+b_{12}) L^{-1}_2G_2((a_{23}+b_{23})G_3(b_{31}u_1)))\\&\quad + G_1((a_{12}+b_{12}) L^{-1}_2G_2(b_{21}u_1)). \end{aligned} \end{aligned}$$

By collecting now the terms with order $\le 0$ we can simplify the previous formula as follows:

$$\begin{aligned} \begin{aligned} u_{1}(t,x)&= {\widetilde{U}}_1^0 + G_1(a_{13}G_3(b_{31}u_1)) + G_1(a_{13}G_3(b_{32}(L^{-1}_2{\widetilde{U}}_2^0)))\\&\quad + G_1(a_{13}G_3b_{32} L^{-1}_2G_2(a_{23}G_3(b_{31}u_1)))\\&\quad + G_1(a_{13}G_3b_{32} L^{-1}_2G_2(b_{23}G_3(b_{31}u_1)))\\&\quad + G_1(a_{13}G_3b_{32}(L^{-1}_2G_2(b_{21}u_1)))\\&\quad + G_1(b_{13}G_3(b_{32} L^{-1}_2G_2(a_{23}G_3(b_{31}u_1))))\\&\quad + G_1(a_{12}L^{-1}_2{\widetilde{U}}_2^0)\\&\quad + G_1(a_{12}L^{-1}_2G_2(a_{23}G_3(b_{31}u_1)))\\&\quad + G_1(a_{12}L^{-1}_2G_2(b_{23})G_3(b_{31}u_1)))\\&\quad + G_1(b_{12}L^{-1}_2G_2((a_{23}G_3(b_{31}u_1)))\\&\quad + G_1(a_{12}L^{-1}_2G_2(b_{21}u_1)) + \text {l.o.t}. \end{aligned} \end{aligned}$$

Looking at the terms

$$\begin{aligned} \begin{aligned}&G_1(a_{13}G_3(b_{32}(L^{-1}_2{\widetilde{U}}_2^0))),\\&G_1(a_{12}L^{-1}_2G_2(b_{21}u_1)),\\&G_1(a_{12}L^{-1}_2G_2(a_{23}G_3(b_{31}u_1))) \end{aligned} \end{aligned}$$

and keeping in mind that in order to get the right Sobolev regularity we need to have operators of order 0, we deduce that $b_{21}$ and $b_{32}$ must have order $-\,1$ while $b_{31}$ must have order $-\,2$. Considering now the initial data

$$\begin{aligned} {\widetilde{U}}_j^0 = U^0_j + G_j((a_{j3}+b_{j3})U^0_3), \quad j=1,2, \end{aligned}$$

by using (17) we obtain

$$\begin{aligned} {\widetilde{U}}_j^0 = U^0_j + G_j((a_{j3}+b_{j3})(G^0_3(u^0_3)+G_3(f_3))), \quad j=1,2. \end{aligned}$$

Combining these formulas with an analysis of the term $G_1(a_{12}L_2^{-1}{\widetilde{U}}_2^0)$ we deduce that ${\widetilde{U}}_2^0$ must belong to $H^{s+1}$. This implies $U_2^0\in H^{s+1}$ and $U_3^0\in H^{s+2}$. Concluding, similarly to the case $m=2$, that is by the Banach fixed point theorem argument on $u_1$ and substitution in $u_2$ and $u_3$, we get anisotropic Sobolev well-posedness by assuming $u^0_1$ and $f_1$ in $H^s$, $u^0_2$ and $f_2$ in $H^{s+1}$, and $u^0_3$ and $f_3$ in $H^{s+2}$. This well-posedness is obtained by means of one invertible operator $L_2$, and in analogy with case $m=2$ the well-posedness can be extended to the whole interval [0, *T*] by an iterated argument. This proves Theorem 1 in the case $m=3$.

### The general case

We are now ready to prove the main result of our paper in the general case of an upper-triangular $m\times m$ matrix, i.e, a matrix *A* of the type

$$\begin{aligned} \begin{bmatrix} \lambda _1(t,x,D_x)&\quad a_{12}(t,x,D_x)&\quad \cdots&\quad a_{1m}(t,x,D_x) \\ 0&\quad \lambda _2(t,x,D_x)&\quad \cdots&\quad a_{2m}(t,x,D_x)\\ \vdots&\quad \vdots&\quad \vdots&\quad \vdots \\ 0&\quad 0&\quad \lambda _{m-1}(t,x,D_x)&\quad a_{m-1 m}(t,x,D_x)\\ 0&\quad 0&\quad \cdots&\quad \lambda _m(t,x,D_x) \end{bmatrix}. \end{aligned}$$

For the convenience of the reader we recall here the statement of Theorem 1.

#### Theorem 1

Let

20 $$\begin{aligned} \left\{ \begin{array}{l@{\quad }l} D_t u = A(t,x,D_x)u + B(t,x,D_x)u + f(t,x), &{}\quad (t,x) \in [0,T] \times \mathbb R^n, \\ \left. u \right| _{t=0} = u_0(x), &{}\quad x \in \mathbb R^n, \end{array} \right. \end{aligned}$$

where $A(t,x,D_x)$ is an upper-triangular matrix of pseudo-differential operators of order 1 in the form (2), and $B(t,x,D_x)$ is a matrix of pseudo-differential operators of order 0, continuous with respect to *t*. Hence, if the lower order terms $b_{ij}$ belong to $C([0,T], \Psi ^{j-i})$ for $i> j$, $u^0_k\in H^{s+k-1}$ and $f_k\in C([0,T],H^{s+k-1})$ for $k=1,\ldots ,m$ then (20) has a unique anisotropic Sobolev solution *u*, i.e., $u_k\in C([0,T], H^{s+k-1})$ for $k=1,\ldots , m$.

#### Proof

Making use of the notations introduced earlier we can write the components of the solution *u* as

21 $$\begin{aligned} u_i(t,x)&= U_i^0 + G_i\left( \sum _{j>i}^m a_{ij}(t,x,D_x)u_j \right) + G_i\left( \sum _{\begin{array}{c} j=1 \\ j \ne i \end{array}}^m b_{ij}(t,x,D_x)u_j \right) \nonumber \\&= U_i^0+\sum _{j<i} G_i (b_{ij}(t,x,D_x)u_j)+\sum _{i<j\le m} G_i((a_{ij}+b_{ij})(t,x,D_x)u_j), \end{aligned}$$

where

$$\begin{aligned} U_i^0 = G_i^0 u_j^0 + G_i(f_i), \end{aligned}$$

and $G_i, G_i^0$ are Fourier integral operator of order 0 for $i=1,\ldots , m$. Note that from the fact that $b_{ij}$ is a symbol of order 0 for every *i*, *j* and, in particular, of order $j-i$ for $j<i$ we obtain that the operator $G_i(b_{ij})$ is of order $j-i$ for $j<i$, while $G_i(a_{ij}+b_{ij})$ is, in general, of order 1. To simplify the argument we introduce the notations $G^{j-i}_{i,j}$ and $G^1_{i,j}$ for the operators $G_i(b_{ij})$ and $G_i(a_{ij}+b_{ij})$, respectively. Here the superscript stands to remind us of the order of the operator. Hence,

$$\begin{aligned} u_i=U_i^0+\sum _{j<i} G^{j-i}_{i,j}(u_j)+\sum _{i<j\le m} G^1_{i,j}(u_j), \end{aligned}$$

for $i=1,\ldots ,m$. By begin by substituting

$$\begin{aligned} u_m=U_m^0+\sum _{j<m} G^{j-m}_{m,j}(u_j), \end{aligned}$$

into

$$\begin{aligned} u_{m-1}=U_{m-1}^0+\sum _{j<m-1} G^{j-m+1}_{m-1,j}(u_j)+G^1_{m-1,m}(u_m). \end{aligned}$$

We get

$$\begin{aligned} \begin{aligned} u_{m-1}&=U_{m-1}^0+\sum _{j<m-1} G^{j-m+1}_{m-1,j}(u_j)+G^1_{m-1,m}U_m^0+\sum _{j<m} G^1_{m-1,m}G^{j-m}_{m,j}(u_j)\\&=(U_{m-1}^0+G^1_{m-1,m}U_m^0)+\sum _{j<m-1} (G^{j-m+1}_{m-1,j}(u_j)+G^1_{m-1,m}G^{j-m}_{m,j}(u_j))\\&\quad + G^1_{m-1,m}G^{-1}_{m,m-1}u_{m-1}. \end{aligned} \end{aligned}$$

Note that it is enough to assume $U^0_m\in H^{s+1}$ and $U^0_{m-1}\in H^s$ to obtain $U_{m-1}^0+G^1_{m-1,m}U_m^0\in H^{s}$. Since all the operators above are of order $\le 0$ we conclude that the operator

$$\begin{aligned} L_{m-1}=I- G^1_{m-1,m}G^{-1}_{m,m-1}:=I-{\mathcal {G}}^0_{m-1} \end{aligned}$$

is invertible on a sufficiently small interval [0, *T*] and, therefore,

22 $$\begin{aligned} u_{m-1}- G^1_{m-1,m}G^{-1}_{m,m-1}u_{m-1}&=(U_{m-1}^0+G^1_{m-1,m}U_m^0)\nonumber \\&\quad +\sum _{j<m-1} (G^{j-m+1}_{m-1,j}(u_j)+G^1_{m-1,m}G^{j-m}_{m,j}(u_j)), \end{aligned}$$

yields

23 $$\begin{aligned} u_{m-1}=L^{-1}_{m-1}{\widetilde{U}}^0_{m-1}+L^{-1}_{m-1}\sum _{j<m-1}{\widetilde{G}}^{j-m+1}_{m-1}u_j, \end{aligned}$$

with ${\widetilde{U}}^0_{m-1}$ and ${\widetilde{G}}^{j-m+1}_{m-1}$ defined by the right-hand side of (22). We now substitute $u_{m}$ and $u_{m-1}$ into $u_{m-2}$ making use of (23). We obtain

24 $$\begin{aligned} u_{m-2}&=U_{m-2}^0+\sum _{j<{m-2}} G^{j-m+2}_{m-2,j}(u_j) \nonumber \\&\quad +G^1_{m-2,m-1}(u_{m-1})+G^1_{m-2,m}(u_{m})\nonumber \\&=U_{m-2}^0+\sum _{j<{m-2}} G^{j-m+2}_{m-2,j}(u_j)+G^1_{m-2,m-1}L^{-1}_{m-1}{\widetilde{U}}^0_{m-1}\nonumber \\&\quad +G^1_{m-2,m-1}L^{-1}_{m-1}\sum _{j< m-2}{\widetilde{G}}^{j-m+1}_{m-1}u_j \nonumber \\&\quad + G^1_{m-2,m-1}L^{-1}_{m-1} {\widetilde{G}}^{-1}_{m-1}u_{m-2} +G^1_{m-2,m}U^0_m \nonumber \\&\quad + G^1_{m-2,m}\sum _{j<m-2} G^{j-m}_{m,j}(u_j)+G^1_{m-2,m}G^{-2}_{m,m-2}u_{m-2}\nonumber \\&\quad +G^1_{m-2,m}G^{-1}_{m,m-1}L^{-1}_{m-1}{\widetilde{U}}^0_{m-1} \nonumber \\&\quad +G^1_{m-2,m}G^{-1}_{m,m-1}L^{-1}_{m-1}\sum _{j<m-2}{\widetilde{G}}^{j-m+1}_{m-1}u_j \nonumber \\&\quad +G^1_{m-2,m}G^{-1}_{m,m-1}L^{-1}_{m-1} {\widetilde{G}}^{-1}_{m-1}u_{m-2}. \end{aligned}$$

We set

25 $$\begin{aligned} {\widetilde{U}}^0_{m-2}&=U_{m-2}^0+G^1_{m-2,m-1}L^{-1}_{m-1}{\widetilde{U}}^0_{m-1}\nonumber \\&\quad +G^1_{m-2,m}U^0_m+G^1_{m-2,m}G^{-1}_{m,m-1}L^{-1}_{m-1}{\widetilde{U}}^0_{m-1}. \end{aligned}$$

The operators $G^1_{m-2,m-1}L^{-1}_{m-1}$ and $G^1_{m-2,m}$ in (25) are of order 1. Keeping in mind that we already assumed $U^0_{m}\in H^{s+1}$ and $U^0_{m-1}\in H^s$, in order to obtain Sobolev order *s* the initial data $U^0_m$, $U^{0}_{m-1}$ and $U^0_{m-2}$ must belong to $H^{s+2}$, $H^{s+1}$ and $H^s$, respectively. Thus,

26 $$\begin{aligned} u_{m-2}= {\widetilde{U}}^0_{m-2}+{\mathcal {G}}^0_{m-2}u_{m-2}+\sum _{j<m-2}{\widetilde{G}}^{j-m+2}_{m-2}u_j, \end{aligned}$$

where ${\mathcal {G}}^0_{m-2}$ is a zero order operator defined by

$$\begin{aligned} \begin{aligned} {\mathcal {G}}^0_{m-2}u_{m-2}&=G^1_{m-2,m-1}L^{-1}_{m-1} {\widetilde{G}}^{-1}_{m-1}u_{m-2}+G^1_{m-2,m}G^{-2}_{m,m-2}u_{m-2}\\&\quad +G^1_{m-2,m}G^{-1}_{m,m-1}L^{-1}_{m-1} {\widetilde{G}}^{-1}_{m-1}u_{m-2}, \end{aligned} \end{aligned}$$

and the last summand in (26) is obtained by collecting all the operators acting on $u_j$ with $j<m-2$ in (24). Since the norm of ${\mathcal {G}}^0_{m-2}$ can be taken strictly less than one in a sufficiently small interval [0, *T*] we have that the operator

$$\begin{aligned} L_{m-2}=I-{\mathcal {G}}^0_{m-2} \end{aligned}$$

is invertible and, therefore,

27 $$\begin{aligned} u_{m-2}=L_{m-2}^{-1} {\widetilde{U}}^0_{m-2} +\sum _{j<m-2}L_{m-2}^{-1}{\widetilde{G}}^{j-m+2}_{m-2}u_j. \end{aligned}$$

Note that ${\widetilde{U}}^0_{m-2} \in H^s$ if $U^0_{m}\in H^{s+2}$, $U^0_{m-1}\in H^{s+1}$ and $U^0_{m-2}\in H^s$. By iterating the same procedure we deduce that

28 $$\begin{aligned} u_{k}= {\widetilde{U}}^0_{k}+{\mathcal {G}}^0_{k}u_k+\sum _{j<k}{\widetilde{G}}^{j-k}_{k}u_j, \end{aligned}$$

where ${\widetilde{U}}^0_k$ depends on $U^0_k$, $U^0_j$ and ${\widetilde{U}}^0_j$ with $j>k$ and ${\mathcal {G}}^0_k$ is a zero order operator defined by using invertible operators $L_{m-1}$, $L_{m-2}$,..., $L_{k}$. In addition, we obtain ${\widetilde{U}}^0_k\in H^s$ since $U^0_m\in H^{s+m-k}$, $U^0_{m-1}\in H^{s+m-k-1},\ldots , U^0_k\in H^s$. It follows that for $k=2$ we have

$$\begin{aligned} u_2={\widetilde{U}}^0_{2}+{\mathcal {G}}^0_{2}u_2+{\widetilde{G}}^{-1}_{2}u_1, \end{aligned}$$

where the operator ${\mathcal {G}}^0_2$ is of zero operator and defined by invertible operators $L_{m-1}, L_{m-2}, \ldots ,L_{2}$, ${\widetilde{G}}^{-1}_{2}$ is of order $-\,1$, and ${\widetilde{U}}^0_2\in H^s$ since $U^0_m \in H^{s+m-2}$, $U^0_{m-1}\in H^{s+m-3},\ldots , U^0_2\in H^s$. Hence, by inverting the operator $L_2=I-{\mathcal {G}}^0_2$ on a sufficiently small interval [0, *T*] we have

$$\begin{aligned} u_2=L^{-1}_2{\widetilde{U}}^0_{2}+L^{-1}_2{\widetilde{G}}^{-1}_{2}u_1. \end{aligned}$$

Now by substitution of $u_2, u_3, \ldots , u_m$ in the equation of $u_1$ we arrive at the formula (28) with $k=1$, i.e.,

$$\begin{aligned} u_1= {\widetilde{U}}^0_{1}+{\mathcal {G}}^0_{1}u_1, \end{aligned}$$

where ${\widetilde{U}}^0_{1}\in H^s$ since $U^0_m\in H^{s+m-1}$, $U^0_{m-1}\in H^{s+m-2},\ldots , U^0_2\in H^{s+1}, U^0_1\in H^s$. Concluding, by the Banach fix point argument we prove that there exists a unique $u_1\in C([0,T], H^s)$ solving the equation above with the given initial conditions. By substitution in the equations for $u_2, \ldots , u_{m-1}, u_m$ we arrive at the desired Sobolev well-posedness with $u_k\in C([0,T], H^{s+k-1})$ for $k=2,\ldots , m$. Note that, since the sufficiently small interval [0, *T*] where we get well-posedness does not depend on the initial data, by a standard iteration argument we can achieve well-posedness on any bounded interval [0, *T*] as stated in the theorem. $\square$

## Schur decomposition of $m \times m$ matrices

In this section we investigate how to reduce an $m\times m$ matrix to the upper triangular form. We recall that such decomposition is well-known for constant matrices and goes under the name of Schur’s triangularisation, with its statement given in Theorem C.

One of the difficulties when dealing with variable multiplicities is the loss of regularity in the parameters at the points of multiplicities. In the following, we will assume that *A* is a matrix of (possibly) complex valued first order symbols, continuous with respect to *t*, i.e., $A(t,x,\xi ) \in \big ( C S^1 \big )^{m \times m}$.

We will now develop a parameter dependent extension of the Schur triangularisation procedure and we will describe it step by step. Then we will give an example for it for the systems of low sizes, namely, for $m=2$ and $m=3$.

In the case of $m=2$ the construction below was introduced in [27] and now we give its general version for systems of any size.

Normal forms of matrices depending on several parameters have a long history and are notoriously involved; for some remarks and related works, we refer the reader to [14, 24, 25, 45].

### First step or Schur step

The first step in our triangularisation follows the construction in the constant case except that we will not get a unitary transformation matrix. For this reason we talk of a Schur step. Throughout this paper $e_i$ denotes the *i*-th vector of the standard basis of $\mathbb R^n$ with an appropriate dimension *n*.

#### Proposition 1

(Schur step) Let the $m \times m$ matrix valued symbol $A(t,x,\xi ) \in (C S^1)^{m \times m}$, have a real eigenvalue $\lambda \in C S^1$ and a corresponding eigenvector $h \in \big ( C S^1 \big )^{m}$ such that there exists $j \in \{ 1, \ldots , m \}$ with

29 $$\begin{aligned} \left\langle h(t,x,\xi ) | e_j \right\rangle \ne 0 \quad \forall (t,x,\xi ) \in [0,T] \times {\mathbb {R}}^n \times \{ |\xi | \ge M \}, \end{aligned}$$

for a sufficiently large $M>0$. Then there exist an $m \times m$ matrix valued symbol $T(t,x,\xi ) (C S^0)^{m \times m}$, invertible for $(t,x,\xi ) \in [0,T] \times {\mathbb {R}}^n \times \{ |\xi | \ge M \}$ with $T^{-1} \in (C S^0)^{m \times m}$, and an $(m-1) \times (m-1)$ matrix valued symbol $E(t,x,\xi ) \in (C S^1)^{m \times m}$, such that

$$\begin{aligned} T^{-1}(t,x,\xi )A(t,x,\xi )T(t,x,\xi ) = \begin{bmatrix} \lambda&\quad a_{12}&\quad \cdots&\quad a_{1m} \\ 0&\quad&\quad&\quad \\ \vdots&\quad&\quad E(t,x,\xi )&\quad \\ 0&\quad&\quad&\quad \end{bmatrix} \end{aligned}$$

for all $$(t,x,\xi ) \in [0,T] \times {\mathbb {R}}^n \times \{ |\xi | \ge M \}$$.

#### Proof

First let us note that we can assume that $j=1$ in (29). If that is not the case, we can exchange the rows 1 and *j* as well as columns 1 and *j* to move the *j*th component of the eigenvector to the first component.

We define the rescaled eigenvector $\mu$ componentwise by

$$\begin{aligned} \mu _{i}(t,x,\xi ) = \frac{\left\langle h(t,x,\xi ) | e_i \right\rangle }{ \left\langle h(t,x,\xi ) | e_1 \right\rangle } \quad \forall i = 1, \ldots , m. \end{aligned}$$

Now we set

$$\begin{aligned} T(t,x,\xi ) = \begin{bmatrix} \mu _{1}&\quad 0&\quad \ldots&\quad 0 \\ \mu _{2}&\quad&\quad&\quad \\ \vdots&\quad&\quad I_{m-1}&\quad \\ \mu _{m}&\quad&\quad&\quad \end{bmatrix}. \end{aligned}$$

Since $\mu _1\equiv 1$ it follows that

$$\begin{aligned} \quad T^{-1}(t,x,\xi ) = \begin{bmatrix} \mu _{1}&\quad 0&\quad \ldots&\quad 0 \\ -\mu _{2}&\quad&\quad&\quad \\ \vdots&\quad&\quad I_{m-1}&\quad \\ -\mu _{m}&\quad&\quad&\quad \end{bmatrix}, \end{aligned}$$

where $I_{m-1}$ is the $(m-1)\times (m-1)$ identity matrix. By direct computations we get

$$\begin{aligned} A T = \begin{bmatrix} \sum \limits _{j=1}^m a_{1j} \mu _{j}&\quad&\quad&\quad \\ \vdots&\quad A_{(2)}&\quad \ldots&\quad A_{(m)}\\ \sum \limits _{j=1}^m a_{mj} \mu _{j}&\quad&\quad&\quad \end{bmatrix} = \begin{bmatrix} \lambda \mu _{1}&\quad&\quad&\quad \\ \vdots&\quad A_{(2)}&\quad \ldots&\quad A_{(m)}\\ \lambda \mu _{m}&\quad&\quad&\quad \end{bmatrix}, \end{aligned}$$

where we used that

30 $$\begin{aligned} \sum _{j=1}^m a_{ij} \mu _{j} = \lambda \mu _{i}, \quad i=1,\ldots ,m, \end{aligned}$$

and denoted the *i*th column of *A* by $A_{(i)}$. The equations in (30) are given by the eigenvalue equation $A \mu = \lambda \mu$. Further, from $\mu _{1} \equiv 1$ we obtain

31 $$\begin{aligned} \nonumber T^{-1} A T= & {} \begin{bmatrix} \lambda \mu _{1}^2&\quad a_{12}\mu _{1}&\quad \ldots&\quad a_{1m}\mu _{1} \\ -\mu _{2}\mu _{1} \lambda + \mu _{2} \lambda&\quad&\quad&\quad \\ \vdots&\quad&\quad E&\quad \\ -\mu _{m} \mu _{1} \lambda + \mu _{m} \lambda&\quad&\quad&\quad&\quad \end{bmatrix} \\= & {} \begin{bmatrix} \lambda&\quad a_{12}&\quad \ldots&\quad a_{1m} \\ 0&\quad&\quad&\quad \\ \vdots&\quad&\quad E&\quad \\ 0&\quad&\quad&\quad&\quad \end{bmatrix}, \end{aligned}$$

which concludes the proof. Note that by construction the matrix *E* has entries in $C S^1$ which depend on *A*. In particular its eigenvalues are the eigenvalues of *A* excluding $\lambda$ (counted as many times as they occur). $\square$

Applying Proposition 1 repeatedly for $m-2$ times to *E*, we obtain a full Schur transformation of *A*, that is a full reduction to an upper triangular form. In the next subsection we describe this iteration in detail. This triangularisation procedure is summarised in Theorem 6 where sufficient conditions on the eigenvectors of *A* are given.

### The triangularisation procedure

The reduction to an upper triangular form or the Schur transformation of *A* is possible under certain conditions on its eigenvectors. More precisely, let

$$\begin{aligned} h_1(t,x,\xi ),\ldots , h_{m-1}(t,x,\xi ) \in \big ( C S^0 \big )^m \end{aligned}$$

be $m-1$ eigenvectors of $A(t,x,\xi ) = [a_{ij}(t,x,\xi )]_{i,j=1}^m$, $a_{ij} \in C S^1$, corresponding to the eigenvalues $\lambda _1(t,x,\xi )$, $\ldots$, $\lambda _{m-1}(t,x,\xi ) \in C S^1$. To formulate the sufficient conditions for the existence of such Schur transformation, we introduce a set of auxiliary vectors $h^{(i)}$, $i=1,\ldots ,m-1$, which depend only on $h_i$ and the previous vectors $h^{(j)} \in C S^0$, $j=1,\ldots ,i-1$. When $i=1$ we set $h^{(1)}=h_1$.

As in Proposition 1 we begin by assuming

32 $$\begin{aligned} \left\langle h^{(1)}(t,x,\xi ) | e_1 \right\rangle \ne 0 \end{aligned}$$

for $$(t,x,\xi ) \in [0,T] \times {\mathbb {R}}^n \times \{ |\xi | \ge M \}$$.

#### Remark 5

As noted in the proof of Proposition 1, we could have that

$$\begin{aligned}\left\langle h^{(1)}(t,x,\xi ) | e_j \right\rangle \ne 0\end{aligned}$$

for another arbitrary $j \in \{ 1, \ldots ,m \}$. Then, we could transform the matrix $A(t,x,\xi )$ by a constant permutation matrix *P* such that $P^{-1}h^{(1)}$ is eigenvector of $P^{-1}AP$ corresponding to $\lambda _1$ which satisfies $\left\langle P^{-1} h^{(1)}(t,x,\xi ) | e_1 \right\rangle \ne 0$. For this reason we state (32) with $h^{(1)}$ and $e_1$.

**Step 1**: By Proposition 1 there exists a matrix $T_1$ such that
  $$\begin{aligned} T^{-1}_1 A T_1 = \begin{bmatrix} \lambda _1&\quad a_{12}&\quad \cdots&\quad a_{1m} \\ 0&\quad&\quad&\quad \\ \vdots&\quad&\quad E_{m-1}&\quad \\ 0&\quad&\quad&\quad \end{bmatrix}. \end{aligned}$$
  The matrix $T_1$ is given by
  $$\begin{aligned} T_1 = \begin{bmatrix} \omega _1&\quad e_2&\quad \ldots&\quad e_m \end{bmatrix}, \quad \omega _1 = \begin{bmatrix} \omega _{11}&\quad \ldots&\quad \omega _{1m} \end{bmatrix}^T \end{aligned}$$
  with
  $$\begin{aligned} \omega _{1j} = \frac{\left\langle h^{(1)}(t,x,\xi ) | e_j \right\rangle }{\left\langle h^{(1)}(t,x,\xi ) | e_1 \right\rangle }. \end{aligned}$$
  In the sequel we make use of the projector $\Pi _k : \mathbb R^m \rightarrow \mathbb R^{m-k}$, $0 \le k \le m-1$, defined by
  $$\begin{aligned} \Pi _k \begin{bmatrix} x_1 \\ \vdots \\ x_m \end{bmatrix} = \begin{bmatrix} x_{k+1} \\ \vdots \\ x_m \end{bmatrix}. \end{aligned}$$
  Note that $\Pi _0$ is the identity map $I_m:\mathbb R^m\rightarrow \mathbb R^m$.
**Step 2**: Since $h_2$ is an eigenvector of *A* with eigenvalue $\lambda _2$ we get that $T_1^{-1}h_2$ is an eigenvector of $T^{-1}_1A T_1$ with eigenvalue $\lambda _2$ as well. By the structure of $T^{-1}_1A T_1$ we easily see that $h^{(2)} := \Pi _1 T_1^{-1} h_2$ is an eigenvector of $E_{m-1}$, corresponding to $\lambda _2$. Arguing as in Remark 5 we assume that
  33 $$\begin{aligned} \left\langle \Pi _1 T_1^{-1} h_2 | e_1 \right\rangle \ne 0 \quad \forall (t,x,\xi ) \in [0,T] \times {\mathbb {R}}^n \times \{ |\xi | \ge M \}, \end{aligned}$$
  to be able to apply Proposition 1 to $E_{m-1}$. We get that there exists an $(m-1) \times (m-1)$ matrix ${\tilde{T}}_2$ such that ${\tilde{T}}_2^{-1}E_{m-1}{\tilde{T}}_2$ is of form
  $$\begin{aligned} \begin{bmatrix} \lambda _2&\quad *&\quad \ldots&\quad *\\ 0&\quad&\quad&\quad \\ \vdots&\quad&\quad E_{m-2}&\quad \\ 0&\quad&\quad&\quad \end{bmatrix}, \end{aligned}$$
  where in the first row the first row of $E_{m-1}$ appears. Thus, setting
  $$\begin{aligned} T_2 = \begin{bmatrix} 1&\quad 0&\quad \ldots&\quad 0 \\ 0&\quad&\quad&\quad \\ \vdots&\quad&\quad {\tilde{T}}_2&\quad \\ 0&\quad&\quad&\quad \end{bmatrix}, \end{aligned}$$
  we obtain
  34 $$\begin{aligned} T_2^{-1}T_1^{-1} A T_1 T_2 = \begin{bmatrix} \lambda _1&\quad *&\quad *&\quad \ldots&\quad *\\ 0&\quad \lambda _2&\quad *&\quad \ldots&\quad *\\ 0&\quad 0&\quad&\quad&\quad \\ \vdots&\quad \vdots&\quad&\quad E_{m-2}&\quad \\ 0&\quad 0&\quad&\quad&\quad \end{bmatrix}. \end{aligned}$$
  Note that in (34) we write explicitly only the entries most relevant to our triangularisation. To compute the matrix ${\tilde{T}}_2$, we set
  $$\begin{aligned} \omega _2 = \begin{bmatrix} \omega _{22}&\ldots&\omega _{2m} \end{bmatrix}^T, \end{aligned}$$
  where
  $$\begin{aligned} \omega _{2j}(t,x,\xi ) := \frac{\left\langle h^{(2)}(t,x,\xi ) | e_j \right\rangle }{\left\langle h^{(2)}(t,x,\xi ) | e_1 \right\rangle } , \quad j=2,\ldots ,m, \end{aligned}$$
  and then
  $$\begin{aligned} {\tilde{T}}_2 = \begin{bmatrix} \omega _2&e_2&\ldots&e_{m-1} \end{bmatrix}. \end{aligned}$$
  It is clear that $T_2$ has the same structure as $T_1$, i.e., it is defined via a rescaled eigenvector as the first column and an identity matrix ($I_{m-1}$ for $T_1$ and $I_{m-2}$ for $T_2$).
**Step K**: By iterating the method $k-1$ times we can find $k-1$ matrices $T_1, T_2, \ldots , T_{k-1}$ of size $m\times m$ such that
  $$\begin{aligned}&T_{k-1}^{-1} \cdot \ldots \cdot T_1^{-1} A T_1 \cdot \ldots \cdot T_{k-1} = \\&\qquad \begin{bmatrix} \lambda _1&\quad *&\quad *&\quad \ldots&\quad \ldots&\quad *\\ 0&\quad \ddots&\quad *&\quad \ldots&\quad \ldots&\quad *\\ 0&\quad 0&\quad \lambda _{k-1}&\quad *&\quad \ldots&\quad *\\ 0&\quad 0&\quad 0&\quad&\quad&\quad \\ \vdots&\quad \vdots&\quad \vdots&\quad&\quad E_{m-{k+1}}&\quad \\ 0&\quad 0&\quad 0&\quad&\quad \end{bmatrix}, \end{aligned}$$
  where $E_{m-k+1}$ is a $(m-k+1) \times (m-k+1)$ matrix and the equality is true on $[0,T]\times {\mathbb {R}}^n\times \{|\xi |\ge M\}$. Since $h_k$ is an eigenvector of *A* corresponding to $\lambda _k$, the vector
  $$\begin{aligned} T_{k-1}^{-1} T_{k-2}^{-1} \cdot \cdots \cdot T_1^{-1} h_k \end{aligned}$$
  is an eigenvector of
  $$\begin{aligned} T_{k-1}^{-1} T_{k-2}^{-1} \cdot \cdots \cdot T_1^{-1} A T_1 T_2 \cdot \cdots \cdot T_{k-1} \end{aligned}$$
  and
  $$\begin{aligned} h^{(k)} := \Pi _{k-1} T_{k-1}^{-1} T_{k-2}^{-1} \cdot \cdots \cdot T_1^{-1} h_k \in \big ( C S^0 \big )^{m-k+1} \end{aligned}$$
  an eigenvector of $E_{m-k+1}$ corresponding to $\lambda _k$. Thus, to satisfy the assumptions of Proposition 1 and keeping in mind Remark 5, we require that
  35 $$\begin{aligned} \left\langle h^{(k)}(t,x,\xi ) | e_1 \right\rangle \ne 0 \quad \forall (t,x,\xi ) \in [0,T] \times {\mathbb {R}}^n \times \{ |\xi | \ge M \}. \end{aligned}$$
  It follows that there exists an $(m-k+1) \times (m-k+1)$ transformation matrix ${\tilde{T}}_k$ such that ${\tilde{T}}^{-1}_{k} \ldots {\tilde{T}}_1^{-1}A{\tilde{T}}_{1} \ldots {\tilde{T}}_k$ is of the form
  $$\begin{aligned} \begin{bmatrix} \lambda _k&\quad *&\quad \ldots&\quad *\\ 0&\quad&\quad&\quad \\ \vdots&\quad&\quad E_{m-k}&\quad \\ 0&\quad&\quad&\quad \end{bmatrix}. \end{aligned}$$
  and set
  $$\begin{aligned} T_k = \begin{bmatrix} I_{k-1}&\mathbf {0} \\ \mathbf {0}&{\tilde{T}}_k \end{bmatrix}. \end{aligned}$$
  The matrix ${\tilde{T}}_k$ is defined by
  $$\begin{aligned} {\tilde{T}}_k = \begin{bmatrix} \omega _k&\quad e_2&\quad \ldots&\quad e_{m-k+1} \end{bmatrix}, \quad \omega _k = \begin{bmatrix} \omega _{kk}&\quad \ldots&\quad \omega _{km} \end{bmatrix}^T, \end{aligned}$$
  where
  $$\begin{aligned} \omega _{kj} = \frac{\left\langle h^{(k)}(t,x,\xi ) | e_j \right\rangle }{\left\langle h^{(k)}(t,x,\xi ) | e_1 \right\rangle }, \quad j = k, \ldots , m. \end{aligned}$$
**Stepm-1**: This is the last step as $E_2$ is a $2 \times 2$ matrix. We have that
  $$\begin{aligned} h^{(m-1)} = \Pi _{m-2} T_{m-2}^{-1} \cdot \cdots \cdot T_{1}^{-1} h_{m-1} \in \big ( C S^0 \big )^{2} \end{aligned}$$
  is an eigenvector of $E_2$ corresponding to $\lambda _{m-1}$ and that ${\tilde{T}}_{m-1}$ exists as before if
  36 $$\begin{aligned} \left\langle h^{(m-1)}(t,x,\xi ) | e_1 \right\rangle \ne 0 \quad \in \forall (t,x,\xi ) \in [0,T] \times {\mathbb {R}}^n \times \{ |\xi | \ge M \}. \end{aligned}$$
  The matrix ${\tilde{T}}_{m-1}$ is given by
  $$\begin{aligned} {\tilde{T}}_{m-1} = \begin{bmatrix} \omega _{m-1}&e_{2} \end{bmatrix} = \begin{bmatrix} \omega _{m-1,m-1}&\quad 0 \\ \omega _{m-1,m}&\quad 1 \end{bmatrix}, \end{aligned}$$
  where
  $$\begin{aligned} \omega _{m-1,j} = \frac{\left\langle h^{(m-1)}(t,x,\xi ) | e_j \right\rangle }{\left\langle h^{(m-1)}(t,x,\xi ) | e_1 \right\rangle } , \quad j =m-1,m, \end{aligned}$$
  and then
  $$\begin{aligned} T_{m-1} = \begin{bmatrix} I_{m-2}&\quad \mathbf 0 \\ \mathbf 0&\quad {\tilde{T}}_{m-1} \end{bmatrix}. \end{aligned}$$

We are now ready to state Theorem 6 which summarises the triangularisation procedure explained above. For the convenience of the reader we recall the notations introduced so far:

- $h_1, \ldots , h_{m-1}$ are the eigenvectors of the matrix *A* corresponding to the eigenvalues $\lambda _1, \ldots , \lambda _{m-1}$.
- $$h^{(1)}=h_1$$ and
  37 $$\begin{aligned} h^{(i)} = \Pi _{i-1}T_{i-1}^{-1} T_{i-2}^{-1} \cdot \,\cdots \, \cdot T_1^{-1} h_i \in \big ( C S^0\big )^{m-k+1} , \end{aligned}$$
  for $$i=2, \ldots ,m-1$$.
- the matrices $T_k$ are inductively defined as follows: $T_0 = I_{m}$ and
  $$\begin{aligned} T_k = \begin{bmatrix} I_{k-1}&\quad \mathbf 0 \\ \mathbf 0&\quad {\tilde{T}}_k \end{bmatrix}, \quad {\tilde{T}}_k = \begin{bmatrix} \omega _{k}&\quad e_{2}&\quad \ldots&\quad e_{m-k} \end{bmatrix}, \quad e_i \in \mathbb R^{m-k}, \end{aligned}$$
  where
  $$\begin{aligned} \omega _{kj} = \frac{\left\langle h^{(k)}(t,x,\xi ) | e_j \right\rangle }{\left\langle h^{(k)}(t,x,\xi ) | e_1 \right\rangle }, \quad j = k,\ldots ,m. \end{aligned}$$

Finally, we note that $h^{(k)}$ depends only on $T_{k-1}$, $\ldots$, $T_1$ and, thus, only on the eigenvectors $h^{(k-1)}$, $\ldots$, $h^{(1)}$.

Summarising, we can formulate a more precise version of Theorem 2.

#### Theorem 6

(Schur Decomposition) Let $A(t,x,\xi ) \in (C S^1)^{m \times m}$ be a matrix with eigenvalues $\lambda _1, \ldots ,\lambda _{m} \in C S^1$, and let $h_1 , \ldots , h_{m-1} \in \big ( C S^0 \big )^m$ be the corresponding eigenvectors. Suppose that for $e_1 \in \mathbb R^{m-i+1}$ the condition

38 $$\begin{aligned} \left\langle h^{(i)}(t,x,\xi ) | e_1 \right\rangle \ne 0, \quad \forall (t,x,\xi ) \in [0,T] \times \mathbb R^n \times \mathbb R^n \end{aligned}$$

holds for all $i=1,\ldots , m-1$, with the notation explained above. Then, there exists a matrix-valued symbol $T(t,x,\xi ) \in (C S^0)^{m \times m}$, invertible for $(t,x,\xi ) \in [0,T] \times \mathbb R^n \times \{ |\xi | \ge M\}$, $T^{-1}(t,x,\xi ) \in (CS^0)^{m\times m}$, such that

$$\begin{aligned} T^{-1}(t,x,\xi )A(t,x,\xi ) T(t,x,\xi ) = \Lambda (t,x,\xi ) + N(t,x,\xi ) \end{aligned}$$

for all $(t,x,\xi ) \in [0,T] \times \mathbb R^n \times \{ |\xi | \ge M \}$, where

$$\begin{aligned} \Lambda (t,x,\xi ) = {{\mathrm{diag}}}(\lambda _1(t,x,\xi ),\lambda _2(t,x,\xi ),\ldots ,\lambda _m(t,x,\xi )) \end{aligned}$$

and

$$\begin{aligned} N(t,x,\xi ) = \begin{bmatrix} 0&\quad N_{12}(t,x,\xi )&\quad N_{13}(t,x,\xi )&\quad \cdots&\quad N_{1m}(t,x,\xi ) \\ 0&\quad 0&\quad N_{23}(t,x,\xi )&\quad \cdots&\quad N_{2m}(t,x,\xi ) \\ \vdots&\quad \vdots&\quad \vdots&\quad \cdots&\quad \vdots \\ 0&\quad 0&\quad 0&\quad \ldots&\quad N_{m-1m}(t,x,\xi )\\ 0&\quad 0&\quad 0&\quad \ldots&\quad 0 \end{bmatrix}, \end{aligned}$$

and *N* is a nilpotent matrix with entries in $C S^1$. Furthermore, the matrix symbol *T* is given by

$$\begin{aligned} T(t,x,\xi ) = T_1 T_2 \cdot \cdots \cdot T_{m-1}, \end{aligned}$$

with the notation explained above.

#### Remark 6

Taking into account Remark 5, let us stress that condition (38) is not restrictive as it can be replaced by the following: suppose that there exist $m-1$ numbers $j_i \in \{ 1,\ldots , m-i+1 \}$, $i=1, \ldots m-1$, such that for all $i=1, \ldots , m-1$

39 $$\begin{aligned} \left\langle h^{(i)}(t,x,\xi ) | e_{j_i} \right\rangle \ne 0 \quad \forall (t,x,\xi ) \in [0,T] \times \mathbb R^n \times \{ |\xi | \ge M \} \end{aligned}$$

holds.

#### Remark 7

If $A(t,x,\xi )$ has complex symbols (as allowed in Theorem 1, see also Remark 1) and real eigenvalues, the eigenvalues of the Schur transformed system clearly remain real. The upper triangular entries may still be complex valued symbols.

#### Remark 8

Theorem 6 is quite general in the sense that the functions $a_{ij}$ could be complex-valued. In this paper, we are concerned with hyperbolic matrices, i.e. we assume that the eigenvalues $\lambda _1, \ldots , \lambda _m$ are real. We stress that the Schur transform does not change the hyperbolicity of the matrix as the eigenvalues of $T^{-1} A T$ are also $\lambda _1, \ldots , \lambda _m$.

#### Remark 9

For our applications in this and future work it is important that the transform *T* in Theorem 6 keeps the regularity of the original matrix *A*, i.e. that the elements of the Schur transform $T^{-1} A T$ are in the same class as the elements of *A*. Here, we stated everything with $C S^1$ and $C S^0$ as that is the regularity considered in this paper. Note that one could replace *C* with $C^k$ or $C^\infty$ and find a matrix *T* such that the transformed matrix $T^{-1}AT$ inherits the same regularity with respect to *t*. In addition, one could also drop the regularity in *t* to $L^\infty$ and the triangularisation procedure would still work preserving the boundedness in *t* through every step.

For the sake of simplicity and the reader’s convenience, in the next subsections we analyse Theorem  6 in the special cases of $m=2$ and $m=3$.

### The case $m=2$

We now formulate Theorem 6 in the special case $m=2$. In this way we recover the formulation given in [27].

#### Theorem 7

([27, Theorem 7.1]) Suppose that $A(t,x,\xi ) \in (CS^1)^{2\times 2}$ admits eigenvalues $\lambda _j(t,x,\xi ) \in C S^1$, $j=1,2$, and an eigenvector $h(t,x,\xi ) \in (C S^0)^2$ satisfying

40 $$\begin{aligned} \left\langle h(t,x,\xi ) | e_j \right\rangle \ne 0, \quad (t,x,\xi ) \in [0,T] \times \mathbb R^n \times \{|\xi | \ge M\}, \end{aligned}$$

for $j=1$ or $j=2$. Then, we can find a $2 \times 2$ matrix valued symbol $T(t,x,\xi ) \in (C S^0)^{2\times 2}$, invertible for $\{|\xi | \ge M \}$ with $T^{-1}(t,x,\xi ) \in (CS^0)^{2\times 2}$, such that

$$\begin{aligned} T^{-1}(t,x,\xi )A(t,x,\xi )T(t,x,\xi ) = \begin{bmatrix} \lambda _1(t,x,\xi )&a_{12}(t,x,\xi ) \\ 0&\lambda _2(t,x,\xi ) \end{bmatrix} \end{aligned}$$

for all $$(t,x,\xi ) \in [0,T] \times \mathbb R^n \times \{ |\xi | \ge M \}$$.

#### Proof

For $2\times 2$ matrices the triangularisation procedure described in the previous subsection can stop at Step 1. By Remark 5, we may assume that (40) holds for the eigenvector *h* corresponding to $\lambda _1$ and for $j=1$. We set $h=h_1$ and $h^{(1)}= h_1$. The vector

$$\begin{aligned} \omega _1 = \begin{bmatrix} \omega _{11}(t,x,\xi ) \\ \omega _{12}(t,x,\xi ) \end{bmatrix}, \quad \quad \omega _{1j}(t,x,\xi ) = \frac{\left\langle h^{(1)}(t,x,\xi ) | e_j \right\rangle }{\left\langle h^{(1)}(t,x,\xi ) | e_1 \right\rangle }, \end{aligned}$$

belongs to $C S^0$ and is an eigenvector of *A* associated to $\lambda _1$. We then set

$$\begin{aligned} T_1(t,x,\xi ) = \begin{bmatrix} \omega _1&\quad e_2 \end{bmatrix} = \begin{bmatrix} \omega _{11}(t,x,\xi )&\quad 0 \\ \omega _{12}(t,x,\xi )&\quad 1 \end{bmatrix}. \end{aligned}$$

With that, we obtain

$$\begin{aligned} A(t,x,\xi ) T_1(t,x,\xi ) = \begin{bmatrix} a_{11}\omega _{11} + a_{12}\omega _{12}&\quad a_{12} \\ a_{21}\omega _{11} + a_{22}\omega _{12}&\quad a_{22} \end{bmatrix} \end{aligned}$$

and finally, with

$$\begin{aligned} T_1^{-1}(t,x,\xi ) = \begin{bmatrix} \omega _{11}(t,x,\xi )&\quad 0 \\ -\omega _{12}(t,x,\xi )&\quad 1 \end{bmatrix}, \end{aligned}$$

we obtain

$$\begin{aligned} \begin{aligned}&T_1^{-1}(t,x,\xi )A(t,x,\xi )T_1(t,x,\xi ) \\&\qquad = \begin{bmatrix} a_{11}\omega _{11}^2+a_{12}\omega _{12}\omega _{11}&\quad a_{12}\omega _{11} \\ -a_{11}\omega _{12}\omega _{11}-a_{12}\omega _{12}^2 + a_{21}\omega _{11} + a_{22}\omega _{12}&\quad -\omega _{12}a_{12}+a_{22} \end{bmatrix} \end{aligned} \end{aligned}$$

By construction, we have

$$\begin{aligned} \begin{aligned}&a_{11}\omega _{11} + a_{12}\omega _{12} = \lambda _1 \omega _{11}, \\&a_{21}\omega _{11} + a_{22}\omega _{12} = \lambda _1 \omega _{12}, \end{aligned} \end{aligned}$$

and $\omega _1=1$. This yields $a_{11}\omega _{11} + a_{12}\omega _{12} = \lambda _1 \omega _{11} = \lambda _1$ and

$$\begin{aligned} \begin{aligned}&-a_{11}\omega _{12}\omega _{11}-a_{12}\omega _{12}^2 + a_{21}\omega _{11} + a_{22}\omega _{12}\\&\qquad = -\omega _{12}(a_{11}\omega _{11}+a_{12}\omega _{12}) + a_{21}\omega _{11} + a_{22}\omega _{12} = -\lambda _1 \omega _{12} + \lambda _1 \omega _{12} = 0. \end{aligned}. \end{aligned}$$

Using $a_{11}+a_{22} = \lambda _1 + \lambda _2$, we obtain

$$\begin{aligned} -\omega _{12}a_{12}+a_{22} = -\omega _{12}a_{12}+a_{22} + a_{11}\omega _{11} - a_{11}\omega _{11} = \lambda _2. \end{aligned}$$

Thus, we get that

$$\begin{aligned} T_1^{-1}(t,x,\xi ) A(t,x,\xi ) T_1(t,x,\xi ) = \begin{bmatrix} \lambda _1(t,x,\xi )&a_{12}(t,x,\xi ) \\ 0&\lambda _2(t,x,\xi ) \end{bmatrix} \end{aligned}$$

for $(t,x,\xi ) \in [0,T] \times \mathbb R^n \times \{ |\xi | \ge M \}$. This concludes the proof. $\square$

#### Example

(i) By direct computations we can easily see that if $h_1=[h_{11}\,\, h_{12}]^T=e_1$ then the matrix *A* is automatically in the upper triangular form. Indeed,
  $$\begin{aligned} a_{21}h_{11}+a_{22}h_{12}=\lambda _1 h_{12} \end{aligned}$$
  implies $a_{21}=0$. A typical example (already discussed in [27]) is the Jordan block matrix
  $$\begin{aligned} A=\begin{bmatrix} 0&\quad 1\\ 0&\quad 0 \end{bmatrix}, \end{aligned}$$
  where $\lambda _1=0$ is an eigenvalue with eigenvector $h_1=e_1$.
(ii) Condition (40) is trivially fulfilled when $\mathrm{det A}\equiv 0$ and *A* is of the form
  $$\begin{aligned} \begin{bmatrix} a&\quad a\\ -a&\quad -a \end{bmatrix}, \end{aligned}$$
  for $a=a(t,x,\xi )$. Indeed, also in this case one can take 0 as an eigenvalue with eigenvector $h_1= [1 \,\, 1]^T$.

### The case $m=3$

With the notation introduced in Sect. 3.2, we assume that the $3 \times 3$ matrix $A(t,x,\xi ) \in (CS^1)^{3 \times 3}$ admits three eigenvalues $\lambda _i(t,x,\xi ) \in C S^1$, $i=1,2,3$, and two corresponding eigenvectors $h_i(t,x,\xi ) \in \big ( C S^0)^3$, $i=1,2$. Then, we set $h^{(1)} := h_1$ and, as in Remark 6 we suppose that there is a $j_1 \in \{1,2,3\}$ with

$$\begin{aligned} \left\langle h^{(1)}(t,x,\xi ) | e_{j_1} \right\rangle \ne 0 \quad \forall (t,x,\xi ) \in [0,T] \times \mathbb R^n \times \{ |\xi | \ge M \}. \end{aligned}$$

Thus, we can set

$$\begin{aligned} \omega _{1j}(t,x,\xi ) = \frac{\left\langle h^{(1)}(t,x,\xi ) | e_j \right\rangle }{\left\langle h^{(1)}(t,x,\xi ) | e_{j_1} \right\rangle }. \end{aligned}$$

Now, we rearrange the matrix *A* such that the first component of $\omega _1$ becomes identically equal to 1. Then, with $j_2,j_3 \in \{ 1,2,3 \} {\setminus } \{ j_1 \}$, we can write

$$\begin{aligned} T_1^{-1} = \begin{bmatrix} \omega _1&e_2&e_3 \end{bmatrix}^{-1} = \begin{bmatrix} \omega _{1j_1}&\quad 0&\quad 0 \\ -\omega _{1j_2}&\quad 1&\quad 0 \\ -\omega _{1j_3}&\quad 0&\quad 1 \end{bmatrix} = \begin{bmatrix} 1&\quad 0&\quad 0 \\ -\omega _{1j_2}&\quad 1&\quad 0 \\ -\omega _{1j_3}&\quad 0&\quad 1 \end{bmatrix}, \end{aligned}$$

which leads to

$$\begin{aligned} T_1^{-1} h_2 = \begin{bmatrix} \omega _{1j_1}&\quad 0&\quad 0 \\ -\omega _{1j_2}&\quad 1&\quad 0 \\ -\omega _{1j_3}&\quad 0&\quad 1 \end{bmatrix} \begin{bmatrix} h_{2j_1} \\ h_{2j_2} \\ h_{2j_3} \end{bmatrix} = \begin{bmatrix} h_{2j_1} \\ -\omega _{1j_2}h_{2j_1} + h_{2j_2} \\ -\omega _{1j_3}h_{2j_1} + h_{2j_3} \end{bmatrix}. \end{aligned}$$

We then get

$$\begin{aligned} h^{(2)} = \Pi _1 T_1^{-1} h_2 = \begin{bmatrix} -\omega _{1j_2}h_{2j_1} + h_{2j_2} \\ -\omega _{1j_3}h_{2j_1} + h_{2j_3} \end{bmatrix} \end{aligned}$$

and the condition (38) that there exists $j \in \{ 1,2 \}$ such that

$$\begin{aligned} \left\langle h^{(2)}(t,x,\xi ) | e_{j} \right\rangle \ne 0 \quad \forall (t,x,\xi ) \in [0,T] \times \mathbb R^n \times \{ |\xi | \ge M \} \end{aligned}$$

translates to: either

41 $$\begin{aligned} -\omega _{1j_2}h_{2j_1} + h_{2j_2} \ne 0 \quad \Rightarrow \quad h_{2j_2} h_{1j_1} - h_{1j_2}h_{2j_1} \ne 0 \end{aligned}$$

or

42 $$\begin{aligned} -\omega _{1j_3}h_{2j_1} + h_{2j_3} \ne 0 \quad \Rightarrow \quad h_{2j_3}h_{1j_1} - h_{1j_3}h_{2j_1} \ne 0 \end{aligned}$$

holds. Thus, assuming that (41) holds, the matrix ${\tilde{T}}_2$ is given by

$$\begin{aligned} \begin{bmatrix} \omega _{21}&\quad 0 \\ \omega _{21}&\quad 1 \end{bmatrix} = \begin{bmatrix} 1&\quad 0 \\ \frac{-\omega _{1j_3}h_{2j_1} + h_{2j_3}}{-\omega _{1j_2}h_{2j_1} + h_{2j_2}}&1 \end{bmatrix}, \quad \omega _{2j} = \frac{\left\langle h^{(2)}(t,x,\xi )|e_j\right\rangle }{\left\langle h^{(2)}(t,x,\xi )|e_{j_2}\right\rangle }, \quad j=1,2, \end{aligned}$$

and the matrix $T_2$ by

$$\begin{aligned} \begin{bmatrix} 1&\quad 0&\quad 0 \\ 0&\quad \omega _{21}&\quad 0 \\ 0&\quad \omega _{22}&\quad 1 \\ \end{bmatrix} = \begin{bmatrix} 1&\quad 0&\quad 0 \\ 0&\quad 1&\quad 0 \\ 0&\quad \frac{-\omega _{1j_3}h_{2j_1} + h_{2j_3}}{-\omega _{1j_2}h_{2j_1} + h_{2j_2}}&\quad 1 \end{bmatrix}. \end{aligned}$$

Thus, we obtain

43 $$\begin{aligned} T(t,x,\xi ) = T_1T_2 = \begin{bmatrix} 1&\quad 0&\quad 0 \\ \omega _{1j_2}&\quad 1&\quad 0 \\ \omega _{1j_3}&\quad 0&\quad 1 \end{bmatrix} \begin{bmatrix} 1&\quad 0&\quad 0 \\ 0&\quad 1&\quad 0 \\ 0&\quad \frac{-\omega _{1j_3}h_{2j_1} + h_{2j_3}}{-\omega _{1j_2}h_{2j_1} + h_{2j_2}}&\quad 1 \end{bmatrix}. \end{aligned}$$

If we have (42) instead of (41), then we would need a permutation matrix

$$\begin{aligned} P_{j_{2} \leftrightarrow j_3} = \begin{bmatrix} 1&\quad 0&\quad 0 \\ 0&\quad 0&\quad 1 \\ 0&\quad 1&\quad 0 \end{bmatrix} \end{aligned}$$

in (43), i.e.

$$\begin{aligned} T(t,x,\xi ) = T_1(t,x,\xi )P_{j_{2} \leftrightarrow j_3} T_2(t,x,\xi ) \end{aligned}$$

and

$$\begin{aligned} T_2(t,x,\xi ) = \begin{bmatrix} 1&\quad 0&\quad 0 \\ 0&\quad 1&\quad 0 \\ 0&\quad \frac{-\omega _{1j_2}h_{2j_1} + h_{2j_2}}{-\omega _{1j_3}h_{2j_1} + h_{2j_3}}&\quad 1 \end{bmatrix}. \end{aligned}$$

See also Remark 6.

Thus, we can state

#### Theorem 8

Suppose that $A(t,x,\xi ) \in (CS^1)^{3 \times 3}$ admits three eigenvalues $\lambda _i \in C S^1$, $i=1,2,3$, and two corresponding eigenvectors $h_i(t,x,\xi ) \in \big ( C S^1 \big )^3$, $i=1,2$. Suppose that there exists a $j_1 \in \{ 1, 2, 3\}$ such that

44 $$\begin{aligned} h_{1j_1}(t,x,\xi ) \ne 0 \quad \forall (t,x,\xi ) \in [0,T] \times \mathbb R^n \times \{ |\xi | \ge M \}. \end{aligned}$$

Further suppose that there exists $j_2 \in \{ 1,2,3\} {\setminus } \{ j_1 \}$ such that

45 $$\begin{aligned} h_{2j_2}h_{1j_1} - h_{1j_2} h_{2j_1} \ne 0 \quad \forall (t,x,\xi ) \in [0,T] \times \mathbb R^n \times \{ |\xi | \ge M \}. \end{aligned}$$

Then, there exists a matrix-valued symbol $T(t,x,\xi ) \in (C S^0)^{3\times 3}$, invertible for all $(t,x,\xi ) \in [0,T] \times \mathbb R^n \times \{ |\xi | \ge M \}$ with $T^{-1}(t,x,\xi ) \in (CS^0)^{3 \times 3}$, such that

$$\begin{aligned} T^{-1}(t,x,\xi )A(t,x,\xi )T(t,x,\xi ) = \Lambda (t,x,\xi ) + N(t,x,\xi ) \end{aligned}$$

holds for all $(t,x,\xi ) \in [0,T] \times \mathbb R^n \times \{ |\xi | \ge M \},$ where $\Lambda (t,x,\xi ) = {{\mathrm{diag}}}(\lambda _1, \lambda _2, \lambda _3)$ and

$$\begin{aligned} N(t,x,\xi ) = \begin{bmatrix} 0&\quad N_{13}(t,x,\xi )&\quad N_{13}(t,x,\xi ) \\ 0&\quad 0&\quad N_{23}(t,x,\xi ) \\ 0&\quad 0&\quad 0 \end{bmatrix}. \end{aligned}$$

We end this subsection by discussing some examples of $3\times 3$ matrices fulfilling the assumptions above on their eigenvalues.

#### Examples

(i) If the matrix *A* has eigenvectors
  $$\begin{aligned} h_1= \begin{bmatrix} 1\\ 0\\ 1 \end{bmatrix} \quad \text {and} \quad h_2= \begin{bmatrix} 1\\ 1\\ 0 \end{bmatrix} \end{aligned}$$
  then conditions (44) and (45) are easily fulfilled with $j_1=1$ and $j_2=2$. Indeed, $h_{11}=1$ and
  $$\begin{aligned} h_{22}h_{11}-h_{12}h_{21}=h_{22}h_{11}=1. \end{aligned}$$
  More in general to satisfy (44) and (45) it would be enough to have two eigenvectors
  $$\begin{aligned} h_1= \begin{bmatrix} h_{11}\\ h_{12}\\ h_{13} \end{bmatrix} \quad \text {and} \quad h_2= \begin{bmatrix} h_{21}\\ h_{22}\\ h_{23} \end{bmatrix} \end{aligned}$$
  with $$h_{11}\ne 0$$, $$h_{22}\ne 0$$ and $$h_{12}=0$$.
(ii) A matrix with eigenvectors
  $$\begin{aligned} h_1= \begin{bmatrix} 1\\ 0\\ 1 \end{bmatrix} \quad \text {and} \quad h_2= \begin{bmatrix} 1\\ 1\\ 0 \end{bmatrix} \end{aligned}$$
  has a special form. Indeed, for $\lambda _1$ and $\lambda _2$ eigenvalues corresponding to $h_1$ and $h_2$, respectively, by using the eigenvector equations we obtain
  $$\begin{aligned} \begin{aligned} a_{13}&=\lambda _1-a_{11},\\ a_{23}&=-a_{21},\\ a_{33}&=\lambda _1-a_{31}, \end{aligned} \end{aligned}$$
  and
  $$\begin{aligned} \begin{aligned} a_{12}&=\lambda _2-a_{11},\\ a_{22}&=\lambda _2-a_{21},\\ a_{32}&=-a_{31}. \end{aligned} \end{aligned}$$
  Hence
  $$\begin{aligned} A=\begin{bmatrix} a_{11}&\quad \lambda _2-a_{11}&\quad \lambda _1-a_{11}\\ a_{21}&\quad \lambda _2-a_{21}&\quad -a_{21}\\ a_{31}&\quad -a_{31}&\quad \lambda _1-a_{31} \end{bmatrix}. \end{aligned}$$
